# The success of community-based management in improving maintenance hemodialysis outcomes: a pilot study

**DOI:** 10.3389/fnut.2025.1652718

**Published:** 2025-09-15

**Authors:** Hui Shi, Lei Fan

**Affiliations:** ^1^Department of Clinical Nutrition, Zibo First Hospital, Zibo, China; ^2^Department of Dialysis Room, Zibo First Hospital, Zibo, China

**Keywords:** nutrition support team, community-based management, maintenance hemodialysis, compliance, mediation effect

## Abstract

**Background and aim:**

Patients on maintenance hemodialysis (MHD) experience various complications, including malnutrition, reduced physical function, and psychological problems. Single-discipline medical approaches prove inadequate in addressing these complex situations. The multidisciplinary management model adopted by the nutritional support team has demonstrated effectiveness in managing such challenges. However, patient compliance remains suboptimal due to limited understanding of treatment regimens, fatigue from prolonged therapy, and insufficient psychological support. Consequently, establishing a patient-centered, transparent, and interactive communication platform is essential to improving treatment adherence through enhanced patient support.

**Methods:**

This prospective randomized controlled trial assigned patients to either an experimental group receiving community-based management or a control group receiving traditional management. Health status was evaluated through laboratory parameters, body composition analysis, anthropometric measurements, and standardized scale assessments.

**Results:**

A total of 28 patients with MHD were enrolled. Four patients died from primary disease (1 in the experimental group and 3 in the control group), leaving 24 who completed the trial. Statistical analysis was conducted on a dataset of 24 patients, including 13 in the experimental group and 11 in the control group. Seven outcomes demonstrated statistically significant differences. In terms of laboratory parameters, the experimental group achieved superior outcomes in serum albumin (12 patients, 92.3% versus 3 patients, 27.3%; *p* = 0.002), hemoglobin (11 patients, 84.6% versus 4 patients, 36.4%; *p* = 0.033), and blood phosphorus levels (10 patients, 76.9% versus 2 patients, 18.2%; *p* = 0.012), compared to control group. Body composition analysis indicated greater improvement in muscle mass (9 patients, 69.2% versus 3 patients, 27.3%; *p* = 0.038) and more favorable visceral fat distribution (11 patients, 84.6% versus 3 patients, 27.3%; *p* = 0.011) in the experimental group. Additionally, the experimental group scored higher on the Short Physical Performance Battery (SPPB; 10 patients, 76.9% versus 3 patients, 27.3%; *p* = 0.038) and exhibited better treatment compliance (10 patients, 76.9% versus 2 patients, 18.2%; *p* = 0.012). Notably, compliance mediated the effect of community-based management on SPPB scores (Proportion Mediated = 76.2%; *p* = 0.038).

**Conclusion:**

Community-based management by the nutrition support team substantially improves patient compliance and enhances clinical outcomes.

**Clinical trial registration:**

chictr.org.cn, identifier ChiCTR2500104523.

## Introduction

Chronic kidney disease (CKD) affects over 10% of the global population (1). As the disease progresses, an increasing number of patients require maintenance hemodialysis (MHD), a trend that continues to rise (2). By 2030, over 5.4 million individuals worldwide are projected to undergo renal replacement therapy (3), with more than 80% of patients with end-stage renal disease (ESRD) dependent on MHD for survival (4, 5). Although MHD sustains life in patients with ESRD, it does not resolve all associated complications. Patients continue to experience malnutrition (6), coronary artery calcification (7), treatment-resistant hypertension (8), frailty (9), impaired physical function (10), and psychological problems (11, 12). The complexity of these conditions limits the effectiveness of single-specialty care in nephrology, highlighting the need for multidisciplinary collaborative management.

The interdisciplinary management model of the nutrition support team (NST) (13) effectively addresses this issue. Established in the 1970s, the NST formed an interdisciplinary team comprising physicians, nurses, nutritionists, and pharmacists (13). Initially, the team aimed to reduce the high rates of central venous catheter-related sepsis and mechanical complications (13). Over time, NST has consistently delivered significant clinical benefits (13). Previously, the NST focused on inpatient care by identifying individuals with nutritional issues, conducting comprehensive nutritional assessments, and delivering safe, effective nutritional interventions (13). Currently, NST provides management for hospitalized patients and extends support to those requiring home-based nutritional therapy (14). Although the NST provides safe and up-to-date nutritional support (15), its impact on clinical outcomes remains poorly understood, with limited supporting evidence (16). Patient compliance remains a critical factor influencing effectiveness (17, 18).

Research highlights a high prevalence of psychological disorders among patients with MHD (11, 12). These conditions substantially reduced both quality of life (19) and self-efficacy (20). According to social cognitive theory, self-efficacy refers to an individual’s confidence in their ability to perform specific behaviors, which is closely linked to treatment compliance (21). Low self-efficacy contributes to poor adherence (20), resulting in biased treatment and uncertain outcomes. For patients with MHD, hemodialysis is only one aspect of treatment; compliance is crucial for achieving effective outcomes.

Community-based management plays a vital role in supporting patient outcomes. Its primary objective is to foster group development by establishing an interactive platform that facilitates patient engagement. Under the guidance of healthcare professionals, patients gain knowledge about their treatment, share personal experiences, ask questions, and collaboratively identify solutions. Regularly structured activities, including health seminars, experience-sharing sessions, and group psychological counseling, encourage proactive self-management. Simultaneously, healthcare providers act as supportive partners, offering essential guidance and assisting patients in addressing specific challenges during therapy, thereby enhancing the overall treatment experience.

## Methods

### Research design

**Figure 1:**
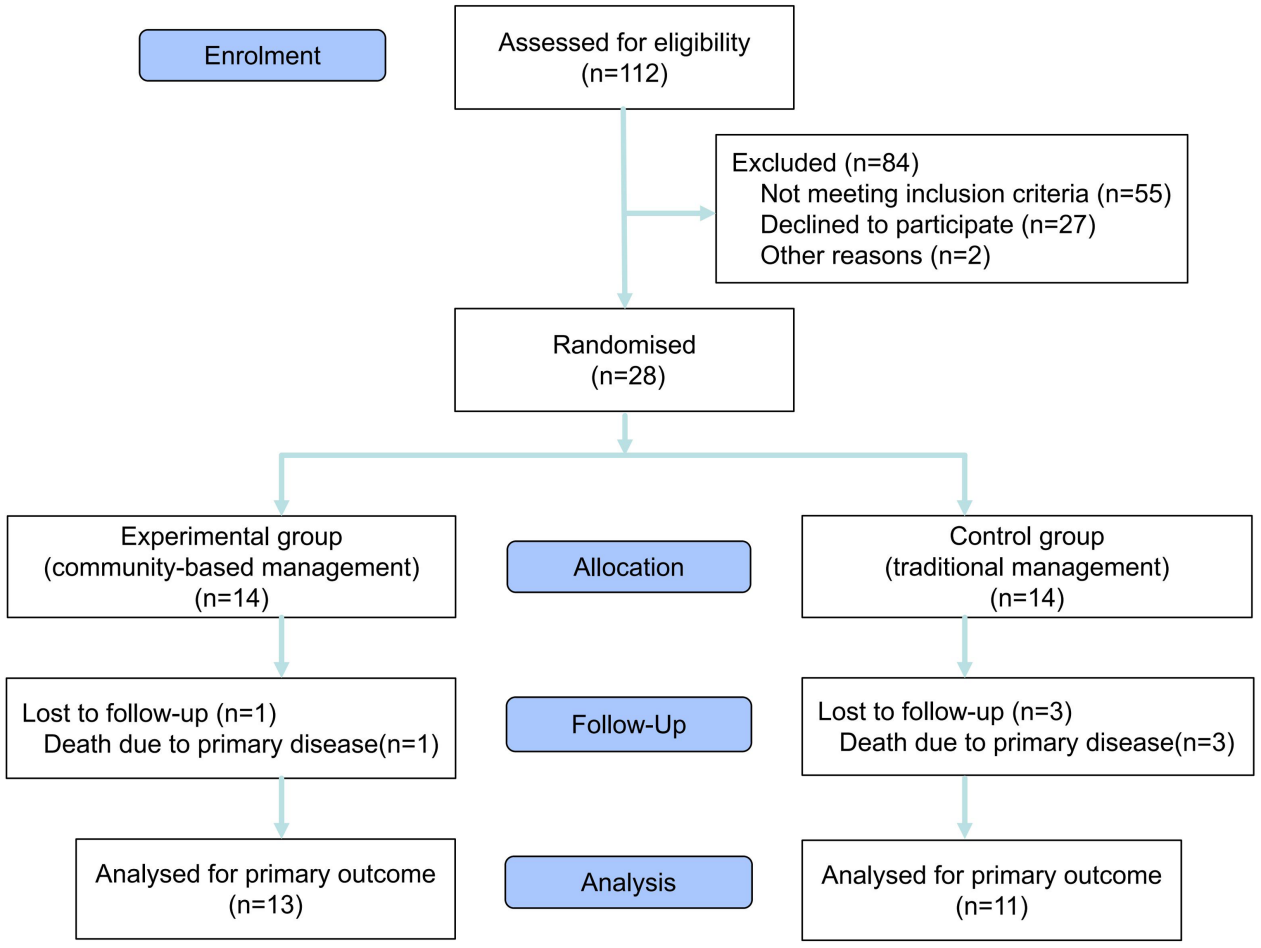
Flowchart for choosing study population.

A prospective randomized controlled trial was conducted in January 2025. This was a single-center pilot study, involving 28 patients recruited from the dialysis department at Zibo First Hospital, with the study period lasting from January to June 2025. Before the trial commenced, demographic and laboratory data were collected for all 112 patients in the dialysis unit. Eligible participants were screened using a baseline survey. The inclusion criteria were as follows: (1) confirmed diagnosis of ESRD; (2) serum albumin (ALB) levels below 38 g/L; (3) absence of malignancy; (4) age ≥ 18 years; (5) provision of informed consent and agreement to comply with all study-related requirements. Patients not meeting these criteria were excluded. Ultimately, 28 patients met eligibility criteria and provided informed consent to participate. Fourteen slips marked with odd numbers and 14 with even numbers were placed in an opaque container. Subsequently, all 28 patients were instructed to randomly draw one slip each from an opaque container. Patients selecting even numbers were assigned to the experimental group, while those drawing odd numbers were allocated to the control group. The experimental cohort adopted a community-based management model. Individualized treatment plans were developed for the 14 participants, incorporating tailored nutritional regimens, structured exercise routines, and prescribed medication schedules. Monthly follow-ups were conducted to adjust treatment protocols as needed. A dedicated WeChat group facilitated communication between researchers and participants, providing weekly online health education, monthly in-person exchange meetings, and twice-monthly group psychological counseling sessions. Themes for all activities were proposed by researchers and communicated to participants 2 days in advance to promote full engagement. Simultaneously, all researchers and participants were notified that they were not allowed to reveal the content of the activities. The control group followed conventional management methods. Individualized treatment plans were also developed for the 14 participants in the control group, comprising nutritional guidance, structured exercise routines, and prescribed medication schedules. Adjustments to treatment plans were made exclusively during monthly follow-ups, with no additional interventions beyond routine care. All 28 participants underwent assessments at both the beginning and end of the trial, including laboratory tests, body composition analysis, anthropometric measurements, and standardized scale evaluation (Figure 1 provides details). This study was conducted as a single-blind trial. To maintain research anonymity and objectivity in data processing, participants’ identities in this study were anonymized through the assignment of randomized numerical identifiers. The experimental data collection was independently executed by a professionally trained medical staff member, ensuring that the entire data acquisition and processing workflow remained confidential from the researchers responsible for statistical analysis.

### Definition of traditional management

Traditional management entails the development of individualized nutrition and exercise regimens, the establishment and maintenance of comprehensive health records, and the scheduling of regular consultations. Management is structured around the individual as the primary unit of care.

### Definition of community-based management

Community-based management involves designing personalized nutrition and exercise regimens, maintaining comprehensive health records, and scheduling regular consultations. In parallel, a WeChat group was established for patients to receive regular-dialysis-related nutritional education and to monitor lifestyle behaviors through a check-in system encompassing diet, physical activity, medication adherence, and related activities. Regular group psychological counseling sessions were conducted, incorporating activities such as mandala painting (22), psychological games, and peer-sharing discussions. Management is structured around the group as the central unit of care.

### Assessment of indicators

#### ALB, prealbumin, and grip strength

Improvement: Values were higher at the end of the trial compared to baseline.

No improvement: (1) Values lower at the end than at baseline; (2) No change.

#### Potassium (K), phosphorus (P), calcium (Ca), hemoglobin (HGB), body fat percentage, visceral fat area, and body mass index

Improvement: (1) Values within the normal range; (2) Values abnormal but trending toward normal.

No improvement: Values were abnormal and trending toward deterioration.

References values were as follows: K 3.5–5.3 mmol/L (23), P 0.85–1.51 mmol/L (24), Ca 2.11–2.52 mmol/L (24), HGB 130–175 g/L for males and 115–150 g/L for females (25), body fat percentage < 17.5% for males and < 31.5% for females (26), visceral fat area < 100 cm^2^ (27), and body mass index 18.5–23.9 kg/m^2^ (28).

#### Creatinine and urea nitrogen

Improvement: Levels were lower at the end compared to the baseline.

No improvement: (1) Levels were higher at the end than at baseline; (2) No change.

#### Muscle mass and arm circumference

Improvement: (1) Levels were higher at the end than at baseline; (2) No change.

No improvement: (1) Levels were lower at the end compared to the baseline.

#### Body water

Improvement: Results reported within the normal range.

No improvement: Results indicated fluid overload or insufficiency.

#### Subjective global assessment (SGA)

Improvement: (1) Grade A at the end of the trial: (2) Improvements from Grade C to B.

No improvement: (1) Grade C at the end; (2) Grade A and B at baseline and Grade B at the end.

SGA interpretation: Grade A indicates normal nutritional status; Grade B represents mild to moderate malnutrition; and Grade C reflects severe malnutrition (29).

#### Sarcopenia risk

Improvement: Sarcopenia five (SARC-F) score < 4 at the end of the trial.

No improvement: Sarcopenia five (SARC-F) score ≥ 4 at the end of the trial.

Evaluation results: according to the SARC-F scale, a score ≥ 4 indicates a risk of sarcopenia (30).

#### Short physical performance battery (SPPB)

Improvement: Score ≥ 10 at the end of the trial.

No improvement: Score < 10 at the end of the trial.

Interpretation: A score ≥ 10 reflects normal physical performance (31).

#### Self-rating depression scale (SDS)

Improvement: Score < 40 at the end of the trial.

No improvement: Score ≥ 40 at the end of the trial.

SDS interpretation: A score ≥ 40 suggests the presence of depressive symptoms (32).

### Statistical analysis

**Figure 2:**
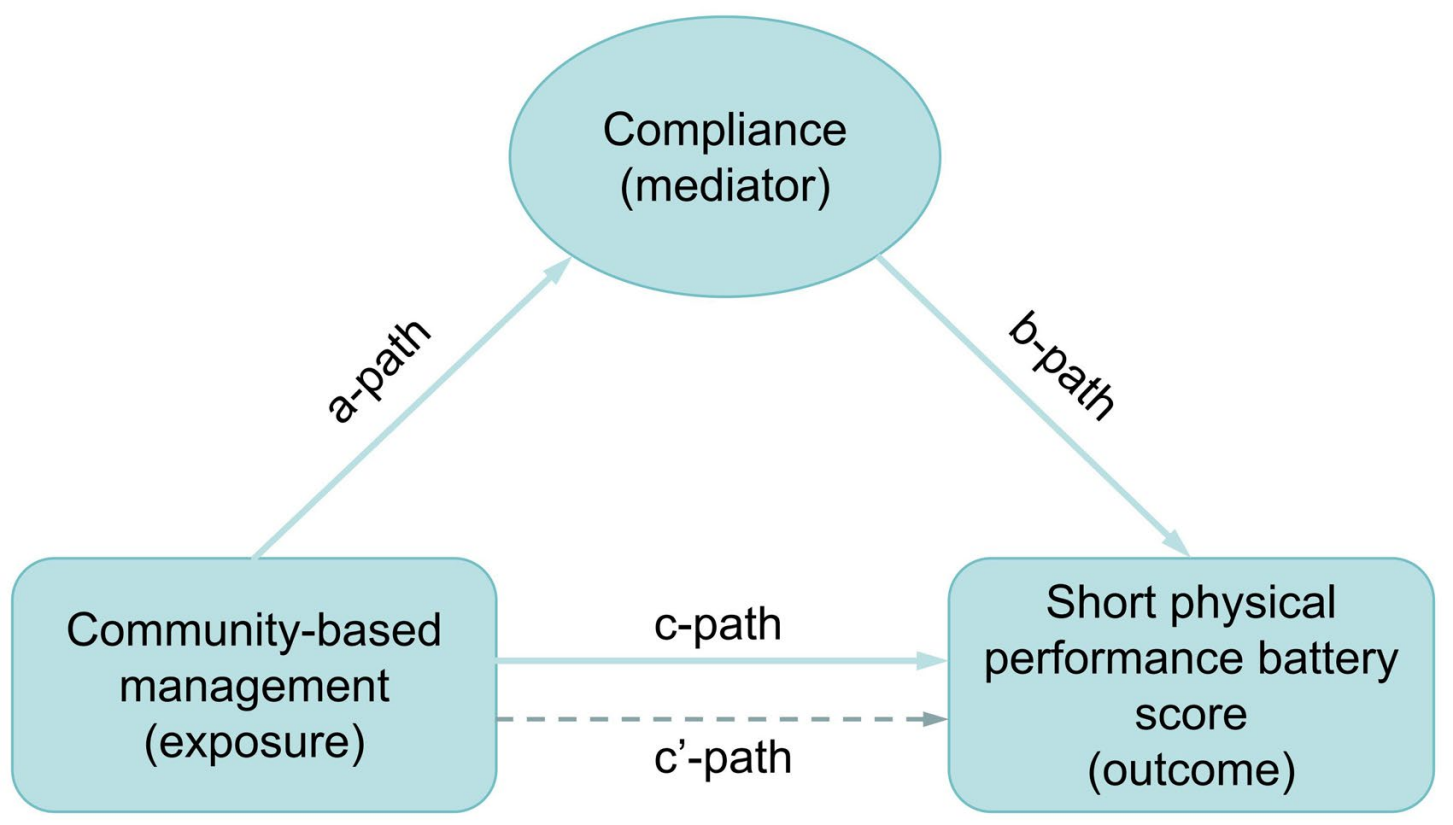
Path diagram of the mediation analysis models.

Data from all 24 patients who completed the trial—13 in the experimental group and 11 in the control group—were included in the statistical analysis. Descriptive analysis was conducted by classifying study variables as either continuous or categorical. Dialysis duration, treated as a continuous variable, is presented as mean and standard deviation. The remaining demographic data and clinical variables were treated as categorical and expressed as frequency and percentage. Statistical differences in categorical variables were assessed using two-sided Fisher’s exact probability tests, while differences in continuous variables were evaluated using the Kruskal–Wallis rank sum test. The management model of the NST was further analyzed using a one-sided Fisher’s exact probability test to assess associations across all outcome measures, including ALB, P, HGB, muscle mass, visceral fat area, SPPB score, and compliance. Statistically significant outcomes (ALB, P, HGB, muscle mass, visceral fat area, and SPPB) were reanalyzed in relation to compliance using a one-sided Fisher’s exact probability test. Compliance was examined as a potential mediator in the relationship between the NST management model and SPPB score. The overall effect of the association between management model (exposure) and SPPB score (outcome) was evaluated through pathway c’. Mediation analysis was conducted through three distinct paths: pathway a evaluated the association between management model and compliance; pathway b assessed the relationship between compliance and SPPB score; and pathway c (direct effect) examined the influence of compliance on the relationship between management model and SPPB score (Figure 2). The mediation effect ratio was calculated as (mediation effect/total effect) × 100. The Karson-Holm-Breen (KHB) method was applied to test the significance of the mediation effect. Furthermore, the impact of demographic factors on compliance was examined. Differences in categorical variables were analyzed using Fisher’s exact probability test, while Kendall’s correlation was used for continuous variables.

All statistical analyses were performed using EmpowerStats (version 2.0) and STATA (version 17.0) software, with a statistical significance defined as *p* < 0.05.

## Results

### Characteristics of participants

Among 24 participants who completed the trial, 12 (50%) were males and 12 (50%) were females. Ten (41.7%) participants were under 60 years of age, while 14 (58.3%) were aged 60 years or older. Two participants (8.3%) were single, and 22 (91.7%) cohabited with a partner. Sixteen (66.7%) participants attained a high school education or above, whereas eight (33.3%) participants did not complete high school. Regarding monthly income, five (20.8%) participants earned less than ¥5,000, and 19 (79.2%) earned more than ¥5,000. Four (16.7%) participants were enrolled in resident medical insurance, while 20 (83.3%) were covered by employee medical insurance. The mean age since initiation of dialysis was 40.7 ± 25.3 months. Utilizing Fisher’s exact probability test, seven indicators exhibited statistically significant differences (*p* < 0.05). These included three laboratory parameters: ALB (*p* = 0.002), P (*p* = 0.012), and HGB (*p* = 0.033); two body composition measures: muscle mass (*p* = 0.038) and visceral fat area (*p* = 0.011); one functional assessment: the SPPB score (*p* = 0.038); and compliance (*p* = 0.012) (Table 1 provides details).

**Table 1:** Characteristics of the study population as categorized according to the management mode.

Characteristic	Total (*n* = 24)	NST management mode		*p*-value
		Traditional management	Community-based management	
		(*n* = 11)	(*n* = 13)	
Gender				1.000
Male	12 (50.0%)	5 (45.5%)	7 (53.8%)	
Female	12 (50.0%)	6 (54.5%)	6 (46.2%)	
Age(year)				1.000
<60	10 (41.7%)	5 (45.5%)	5 (38.5%)	
≥60	14 (58.3%)	6 (54.5%)	8 (61.5%)	
Marriage				1.000
Single	2 (8.3%)	1 (9.1%)	1 (7.7%)	
Married	22 (91.7%)	10 (90.9%)	12 (92.3%)	
Education				0.390
<High school	8 (33.3%)	5 (45.5%)	3 (23.1%)	
≥High school	16 (66.7%)	6 (54.5%)	10 (76.9%)	
Income per month(¥)				0.630
<5,000	5 (20.8%)	3 (27.3%)	2 (15.4%)	
≥5,000	19 (79.2%)	8 (72.7%)	11 (84.6%)	
Medical insurance				0.300
Resident medical insurance	4 (16.7%)	3 (27.3%)	1 (7.7%)	
Employee medical insurance	20 (83.3%)	8 (72.7%)	12 (92.3%)	
Dialysis age(month)	40.7 ± 25.3	45.6 ± 27.9	36.5 ± 23.1	0.562*
ALB				0.002
No improvement	9 (37.5%)	8 (72.7%)	1 (7.7%)	
Improvement	15 (62.5%)	3 (27.3%)	12 (92.3%)	
PA				0.123
No improvement	13 (54.2%)	8 (72.7%)	5 (38.5%)	
Improvement	11 (45.8%)	3 (27.3%)	8 (61.5%)	
K				0.357
No improvement	6 (25.0%)	4 (36.4%)	2 (15.4%)	
Improvement	18 (75.0%)	7 (63.6%)	11 (84.6%)	
P				0.012
No improvement	12 (50.0%)	9 (81.8%)	3 (23.1%)	
Improvement	12 (50.0%)	2 (18.2%)	10 (76.9%)	
Ca				0.300
No improvement	4 (16.7%)	3 (27.3%)	1 (7.7%)	
Improvement	20 (83.3%)	8 (72.7%)	12 (92.3%)	
Cr				0.659
No improvement	7 (29.2%)	4 (36.4%)	3 (23.1%)	
Improvement	17 (70.8%)	7 (63.6%)	10 (76.9%)	
UN				1.000
No improvement	6 (25.0%)	3 (27.3%)	3 (23.1%)	
Improvement	18 (75.0%)	8 (72.7%)	10 (76.9%)	
HGB				0.033
No improvement	9 (37.5%)	7 (63.6%)	2 (15.4%)	
Improvement	15 (62.5%)	4 (36.4%)	11 (84.6%)	
Muscle mass				0.038
No improvement	12 (50.0%)	8 (72.7%)	4 (30.8%)	
Improvement	12 (50.0%)	3 (27.3%)	9 (69.2%)	
Body fat percentage				1.000
No improvement	6 (25.0%)	3 (27.3%)	3 (23.1%)	
Improvement	18 (75.0%)	8 (72.7%)	10 (76.9%)	
Visceral fat area				0.011
No improvement	10 (41.7%)	8 (72.7%)	2 (15.4%)	
Improvement	14 (58.3%)	3 (27.3%)	11 (84.6%)	
Body water				0.182
No improvement	7 (29.2%)	5 (45.5%)	2 (15.4%)	
Improvement	17 (70.8%)	6 (54.5%)	11 (84.6%)	
Body mass index				0.576
No improvement	3 (12.5%)	2 (18.2%)	1 (7.7%)	
Improvement	21 (87.5%)	9 (81.8%)	12 (92.3%)	
Grip strength				0.206
No improvement	9 (37.5%)	6 (54.5%)	3 (23.1%)	
Improvement	15 (62.5%)	5 (45.5%)	10 (76.9%)	
Arm circumference				0.123
No improvement	13 (54.2%)	8 (72.7%)	5 (38.5%)	
Improvement	11 (45.8%)	3 (27.3%)	8 (61.5%)	
SGA				0.206
No improvement	9 (37.5%)	6 (54.5%)	3 (23.1%)	
Improvement	15 (62.5%)	5 (45.5%)	10 (76.9%)	
SARC-F				0.142
<4	19 (79.2%)	7 (63.6%)	12 (92.3%)	
≥4	5 (20.8%)	4 (36.4%)	1 (7.7%)	
SPPB				0.038
<10	11 (45.8%)	8 (72.7%)	3 (23.1%)	
≥10	13 (54.2%)	3 (27.3%)	10 (76.9%)	
SDS				0.390
<40	16 (66.7%)	6 (54.5%)	10 (76.9%)	
≥40	8 (33.3%)	5 (45.5%)	3 (23.1%)	
Compliance				0.012
Partial implemented and rejected	12 (50.0%)	9 (81.8%)	3 (23.1%)	
Fully implemented	12 (50.0%)	2 (18.2%)	10 (76.9%)	

NST, nutrition support team; ALB, albumin; PA, prealbumin; K, potassium; P, phosphorus; Ca, calcium; Cr, creatinine; UN, urea nitrogen; HGB, hemoglobin; SGA, subjective global assessment; SARC-F, sarcopenia five; SPPB, short physical performance battery; SDS, self-rating depression scale; Categorical variables are shown as *N* (%), while continuous variables are shown as Mean ± SD; *p*-values for categorical variables are determined using Fisher’s exact probability test, while for continuous variables (*), the Kruskal-Wallis test is used.

### Correlation between NST management model and seven statistically significant outcomes

A one-sided Fisher’s exact probability test indicated improvement across all seven indicators in the community-based management group (Table 2).

**Table 2:** Correlation of NST’s management mode with seven statistically significant outcomes.

Statistically significant outcomes	ALB	P	HGB	Muscle mass	Visceral fat area	SPPB	Compliance
*p*-value	0.002	0.006	0.021	0.021	0.007	0.021	0.006

NST, nutrition support team; ALB, albumin; P, phosphorus; HGB, hemoglobin; SPPB, short physical performance battery; *p*-values are derived from a one-sided Fisher’s exact probability test.

### Correlation between compliance and six outcomes: ALB, P, HGB, muscle mass, visceral fat area, and SPPB

A one-sided Fisher’s exact probability test identified a significant association between compliance and SPPB scores (*p* < 0.001), indicating that participants with higher compliance achieved SPPB scores ≥ 10 (Table 3).

**Table 3:** Correlation between compliance and six outcomes, including ALB, P, HGB, Muscle mass, Visceral fat area and SPPB.

Statistically significant outcomes	ALB	P	HGB	Muscle mass	Visceral fat area	SPPB
*p*-value	0.200	0.110	0.200	0.207	0.107	<0.001

ALB, albumin; P, phosphorus; HGB, hemoglobin; SPPB, short physical performance battery; *p*-values are derived from a one-sided Fisher’s exact probability test.

### Analysis of mediation effects between NST community-based management and SPPB score

Compliance significantly mediated the effect of NST community-based management on the SPPB score, accounting for 76.2% of the total effect (*p* = 0.038) (Table 4).

**Table 4:** Analysis of mediation effects between NST’s community-based management and SPPB score.

Analysis of mediation effects	Exposure: community-based management	Mediator: compliance	Outcome: SPPB score	Proportion mediated (%)
	Direct effect	Mediator effect	Total effect	
Coefficient	0.676	2.156	2.832	76.2
*p*-value	0.620	0.038	0.028	

NST, nutrition support team; SPPB, short physical performance battery.

### Association between compliance and demographic indicators

Fisher’s exact probability test and Kendall correlation identified a significant association between compliance and education level, with participants possessing higher educational attainment demonstrating superior compliance (*p* = 0.014) (Table 5).

**Table 5:** Relationship between compliance and demographic indicators.

Demographic indicators	Gender	Age	Marriage	Education	Income per month	Medical insurance	Dialysis age
*p*-value	0.684	0.680	1.000	0.014*	0.317	0.590	0.436**

*p*-value is derived from the two-sided Fisher’s exact probability test; * *p*-value is obtained from the one-sided Fisher’s exact probability test, and ** *p*-value is calculated using Kendall’s correlation.

## Discussion

In the 21st century, chronic diseases have emerged as the predominant global health concern (33). Approximately 2% of patients with CKD progress to ESRD annually (34, 35). As a principal therapy for ESRD, MHD requires prolonged and frequent sessions and carries substantial complications. Prolonged dialysis exacerbates malnutrition (36), frailty (37), diminished physical function (38, 39), reduced quality of life (19), and psychological problems (11, 12). The prognosis remains poor; despite advancements in dialysis techniques and improvements in care quality, the average life expectancy of patients undergoing dialysis is nearly half that of age-matched individuals in the general population (40). The five-year survival rate after initiating maintenance dialysis was only 40% (41). Notably, MHD adversely influences nutritional status (42), physical capabilities (41), and psychology well-being (11, 12).

Malnutrition is prevalent among dialysis patients (36) and is driven by numerous risk factors, including uremia, dialysis-related complications, inflammation, acidosis, endocrine disorders, nutrient loss during treatment, psychological conditions, reduced physical activity, intestinal dysbiosis, and anorexia (6, 43). In patients with ESRD, malnutrition is associated with diminished quality of life and increased mortality risk (6, 44). Therefore, implementation of preventive and targeted nutritional interventions remains critical for addressing malnutrition. Evidence from existing studies demonstrates that nutrition-focused strategies, particularly those involving dietitian guidance, considerably improve nutritional status and physical function in patients (45–47). However, hyperphosphatemia continues to present clinical challenges (48). In this study, the community-based management group achieved superior control of serum P levels compared to the traditional management group (10 patients, 76.9% versus 2 patients, 18.2%; *p* = 0.012), highlighting the effectiveness of the community-based management model. The integration of nutritional interventions within this framework contributed to enhanced outcomes. Notably, greater improvements in ALB levels (12 patients, 92.3% versus 3 patients, 27.3%, *p* = 0.002) and HGB levels (11 patients, 84.6% versus 4 patients, 36.4%, *p* = 0.033) were observed in the community-based management group compared to the traditional management group, further supporting the clinical benefits of this approach.

Frailty is prevalent among patients undergoing MHD, resulting in increased physical vulnerability and reduced physiological resilience (49). This condition impairs self-care capacity, lowers quality of life, and elevates the risk of adverse events such as falls and fractures (9). Exercise participation can mitigate physical weakness and improve cardiovascular function, functional capacity, and overall quality of life (50). Evidence indicates that appropriately prescribed exercise provides significant clinical benefits for this population (51, 52). Engagement in exercise was associated with alleviation of depressive symptoms (53), improved sleep quality (54), and reduced risk of cardiovascular disease (55). This study’s findings are consistent with previous studies demonstrating greater improvements in body composition and physical function in the community-based management group compared to the traditional management group. Notably, increases in muscle mass were more pronounced in the community-based management group (9 patients, 69.2% versus 3 patients, 27.3%; *p* = 0.038), and visceral fat levels were more favorable (11 patients, 84.6% versus 3 patients, 27.3%; *p* = 0.011). Regarding the SPPB score, the community-based management group exhibited superior performance (10 patients, 76.9% versus 3 patients, 27.3%; *p* = 0.038), supporting the efficacy of the community-based management model. Furthermore, analysis of patient compliance indicated that the improvement observed in the community-based management group (10 group, 76.9% versus 2 patients, 18.2%, *p* = 0.012) was not solely attributable to the intervention but also served as a significant mediator in the enhancement of SPPB score (Proportion Mediated = 76.2%; *p* = 0.038).

Psychological disorders are highly prevalent among patients undergoing MHD, with the majority experiencing anxiety, depression, and various forms of psychological distress (11, 12). These symptoms are occasionally atypical and frequently underdiagnosed (56), yet the associated disease burden remains substantial (57). Recent research has identified potential interventions for this issue. Notably, a study on guided meditation reported significant improvements in self-confidence, psychological well-being, and reduced perceived stress among participants (58). A separate investigation on psychoeducation demonstrated that effective psychoeducational strategies significantly improved psychological symptoms and enhanced quality of life in both the short- and medium-term durations (59). However, this study did not provide direct evidence regarding the impact of community-based management models on depressive symptoms, potentially due to limitations imposed by a small sample size. Nevertheless, a rationale persists to suggest that the psychological support component of the community-based management model contributed to improved outcomes, as indicated by the significantly higher compliance rates in the community-based management group (10 patients, 76.9% versus 2 patients, 18.2%, *p* = 0.012). As previously discussed, compliance serves as an indicator of self-efficacy, which correlates positively with self-confidence (21).

Given the multifaceted nature of diseases and health challenges faced by patients with MHD, the effective application of health management strategies has become a critical focus. Contemporary medical practice in the 21st century is guided by three core principles: patient-centered care, active involvement of patients and their families, and shared decision-making (60). The emphasis in health management has shifted from medical specialist guidance to active patient engagement. In the context of chronic dialysis treatment, patient-reported outcomes should be prioritized over evidence-based clinical indicators (61). Extensive research by prominent scholars and organizations has contributed to advancing self-management among dialysis patients (62–64), yielding significant findings. However, not all studies have yielded favorable outcomes (65). Non-disease-related factors, such as public awareness of dialysis (66), patient-incurred expenses (67), and insurance and reimbursement policies (67), also influence the implementation of health management strategies. For instance, this study found that patients with higher educational attainment exhibited significantly superior compliance (*p* = 0.014).

In conclusion, the burden of disease and health-related challenges among patients with MHD is multifaceted, and as research progresses, increasingly complex issues are anticipated. As an emerging discipline, NST has demonstrated considerable potential in nutritional management and continues to develop (17); however, limited research has specifically explored its application in MHD. The community-based management model utilized in this study prioritizes team-based care and psychological counseling, aligning with the medical philosophies of “patient-centered care” (60).

## Conclusion

The community-based management model led by the NST improves patient compliance and delivers greater clinical benefits.

### Strengths of this study

This study introduced an innovative community-based management model that designates the patient group as the primary unit of care. It emphasizes team-based collaboration and psychological support while encouraging patients to engage in proactive self-management. This study is grounded in the principle of “patient first, with medical personnel in a supportive role,” aligning with contemporary medical philosophy. It also highlights the concept that “patient behavior is a key determinant of prognosis.”

### Limitations of this study

This single-center trial involved a limited sample size, introducing an inherent risk of bias in the findings. Such bias is a common concern in small-sample research. Additional studies with similar designs are needed to consistently validate these findings. Although the nutritional and exercise protocols were designed and supervised by medical professionals, their patient-led implementation may have introduced subjective bias that is difficult to mitigate. Furthermore, while data from deceased patients were excluded from the statistical analysis, fluctuations in the sample sizes of the two groups during the study period may have influenced the results.

## Data Availability

The original contributions presented in the study are included in the article/Supplementary material, further inquiries can be directed to the corresponding author.
